# The *OSU1/QUA2/TSD2*-Encoded Putative Methyltransferase Is a Critical Modulator of Carbon and Nitrogen Nutrient Balance Response in *Arabidopsis*

**DOI:** 10.1371/journal.pone.0001387

**Published:** 2008-01-02

**Authors:** Peng Gao, Zeyu Xin, Zhi-Liang Zheng

**Affiliations:** 1 Department of Biological Sciences, Lehman College, City University of New York, Bronx, New York, United States of America; 2 Plant Sciences PhD Subprogram, Graduate School and University Center, City University of New York, New York, New York, United States of America; University of California at Davis, United States of America

## Abstract

The balance between carbon (C) and nitrogen (N) nutrients must be tightly coordinated so that cells can optimize their opportunity for metabolism, growth and development. However, the C and N nutrient balance perception and signaling mechanism remains poorly understood. Here, we report the isolation and characterization of two allelic *oversensitive to sugar1* mutants (*osu1-1, osu1-2*) in *Arabidopsis thaliana*. Using the cotyledon anthocyanin accumulation and root growth inhibition assays, we show that the *osu1* mutants are more sensitive than wild-type to both of the imbalanced C/N conditions, high C/low N and low C/high N. However, under the balanced C/N conditions (low C/low N or high C/high N), the *osu1* mutants have similar anthocyanin levels and root lengths as wild-type. Consistently, the genes encoding two MYB transcription factors (MYB75 and MYB90) and an Asn synthetase isoform (ASN1) are strongly up-regulated by the *OSU1* mutation in response to high C/low N and low C/high N, respectively. Furthermore, the enhanced sensitivity of *osu1-1* to high C/low N with respect to anthocyanin accumulation but not root growth inhibition can be suppressed by co-suppression of *MYB75*, indicating that MYB75 acts downstream of OSU1 in the high C/low N imbalance response. Map-based cloning reveals that *OSU1* encodes a member of a large family of putative methyltransferases and is allelic to the recently reported *QUA2/TSD2* locus identified in genetic screens for cell-adhesion-defective mutants. Accumulation of *OSU1/QUA2/TSD2* transcript was not regulated by C and N balance, but the *OSU1* promoter was slightly more active in the vascular system. Taken together, our results show that the *OSU1/QUA2/TSD2*-encoded putative methyltransferase is required for normal C/N nutrient balance response in plants.

## Introduction

The tight coordination of cellular carbon (C) and nitrogen (N) metabolism must be accomplished for proper growth and development. Carbohydrates provide both the energy and the carbon-skeletons used for ammonium assimilation during amino acid biosynthesis, while amino acids and proteins are the key building blocks for the cell [1], [2]. Therefore, cells must monitor both the status of C and N nutrients and the balance between C and N to optimize their opportunity for metabolism, growth and development.

Despite numerous studies on the sensing and signaling mechanisms for levels of available C (such as glucose and sucrose) or N (such as nitrate, ammonium, and amino acids), the interaction between C and N has received attention only recently [1]–[8]. In bacteria, the PII protein has been shown to be a central processor for integrating N and C metabolism [4]. In yeast or animal systems, Ras/Rho GTPases, Snf1/AMPK or the “hexosamine” signaling pathway and other components are involved in sensing and signaling the glucose or amino acid status [4], [9], [10]. Surprisingly, not much is known about the cross-talk between C and N signaling pathways, although a C and N nutrient coincidence detection system exists (such as reported in [11]). Some examples of the cross-talk include the convergence of Snf1-glucose and TOR-nitrogen signaling pathways onto the Gln3 transcription factor [12], and the TOR pathway-mediated carbon catabolite repression of amino acid permeases [13].

The molecular mechanisms by which plants sense the C and N balance or the C∶N ratio and transduce the signal remain even more poorly understood [1], [2]. Most plants are autotrophic organisms that synthesize both carbohydrates and amino acids and monitor their C and N status. There has been some exciting progress in understanding the mechanisms of sugar signaling [14]–[16] and the regulation of nitrogen metabolism/signaling [17]–[19]. Recent DNA microarray studies have also shown that many genes are regulated by C alone, N alone, or their interactions, but only a limited number of genes have been shown to be regulated by the C∶N ratio [7], [8], [20]–[25]. These genes are mostly involved in C or N metabolism such as Asn and Gln synthetase and Glu dehydrogenase genes [21]–[23], [26]–[28] or transport such as a nitrate transporter gene [3], [29]. On the other hand, studies on plant orthologs of bacterial PII and mammalian TOR have not provided convincing support for their involvement in C/N balance signaling in plants. For example, the *Arabidopsis* ortholog of PII, GLB1, was implicated in the C∶N ratio sensing, based on an ectopic overexpression study [30]. Although plant PII orthologs were speculated to function as a sensor for both C and N [31], the T-DNA knockout mutants of *GLB1* exhibit only weak phenotypes [32]. This indicates a subtle involvement of the PII protein in the regulation of some steps of primary C and N metabolism in plants [32]. The TOR ortholog has not been suggested to be involved in vegetative development [33], and the functions of its regulators (such as RAPTOR1A and RAPTOR1B) in nutrient signaling have not been reported [34], [35]. The molecular mechanism underlying the perception or signaling of the C and N balance in plants remains a mystery.

Here, we describe the isolation of the *oversensitive to sugar1* (*osu1)* mutants. Using a modified C/N balance bioassay, we show that they are hypersensitive to imbalanced C/N (both high C/low N and low C/high N) but are similar to the wild-type (WT) when C/N ratios are more balanced (low C/low N, or high C/high N). Furthermore, *MYB75*, a transcription factor gene that is up-regulated by the *OSU1* mutations, acts downstream of *OSU1* in response to high C/low N. *OSU1* suppresses the low C/high N-activation of an Asn synthetase gene (*ASN1*) which is known to be transcriptionally regulated by the C and N balance. Molecular cloning reveals that *OSU1* encodes a putative methyltransferase that belongs to a functionally uncharacterized family with a total of 29 members in the Arabidopsis genome. While we were preparing this manuscript, two reports showed that the allelic mutations in *OSU1* (called *QUA2/TSD2*) affect cell adhesion and that QUA2/TSD2 is localized in the Golgi [36], [37]. *OSU1/QUA2/TSD2* has a slight preference for expression in the vascular system, consistent with its role in nutrient response. Taken together, our results show that the *OSU1/QUA2/TSD2*-encoded putative methyltransferase modulates the C and N balance response in *Arabidopsis*.

## Results

### Isolation of the *osu1* mutants

**Figure 1:**
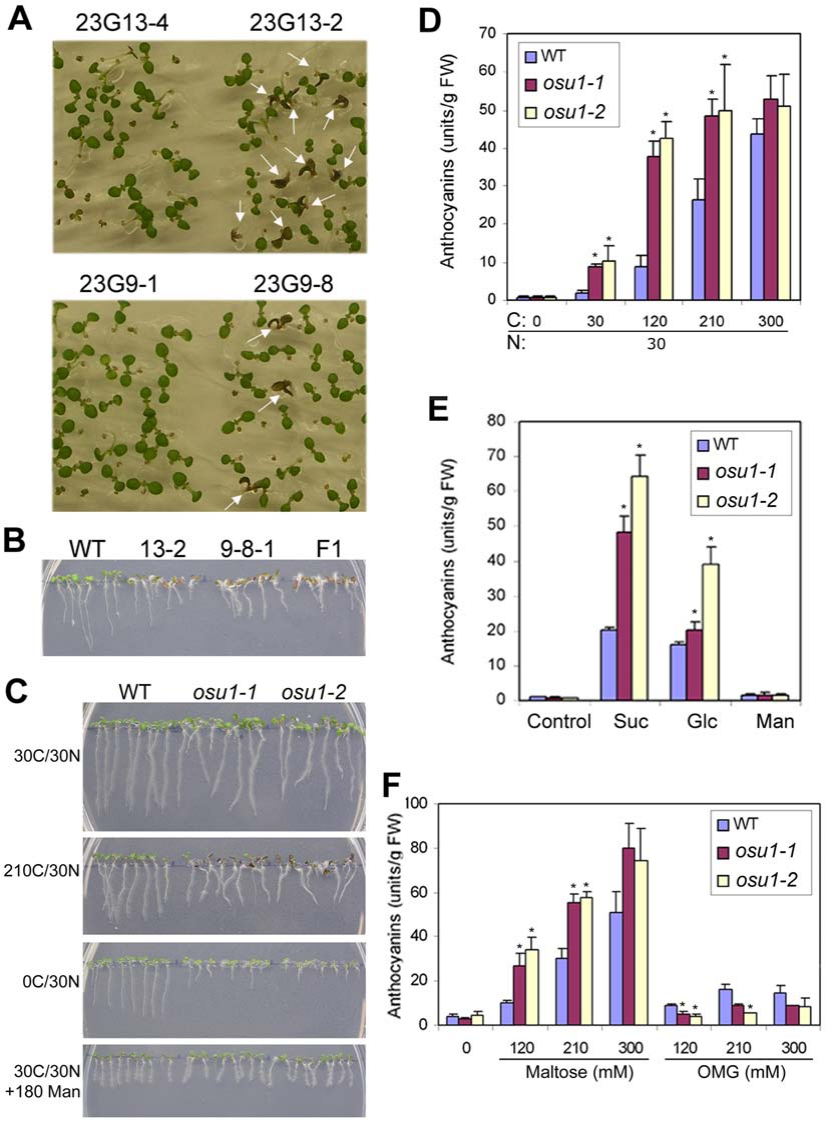
Isolation and characterization of the *osu1* mutants. Seedlings shown in this figure were all grown in the commercially prepared 1/2 MS medium which contained 30 mM total N (30N) for seven days. For (D-F), the average of anthocyanin contents from three replicates, each with 8–10 seedlings, is shown, with the bar representing the SD. Statistical analysis by one way ANOVA; the asterisk (*) above the column indicates a significant difference (p<0.05) between *osu1-1* or *osu1-2* and wild-type (WT) under the same nutrient condition. (A) T3 seedlings of the two *P_ROP2_:GUS* lines (23G13-2 and 23G9-8) exhibited segregation of reddish purple cotyledons (indicated by arrows) and normal green cotyledons when grown in the presence of 1% sucrose (approximately 30 mM Suc, designated 30C) and 50 µg/mL hygromycin. Other T3 seedlings from the two parental plants (23G13-4 and 23G9-1) did not show such a phenotype. (B) Failure of 23G13-2 to complement 23G9-8-1 under 120 mM sucrose. 13-2 represents 23G13-2, which is designated *osu1-1*; 9-8-1 represents 23G-9-8-1, which is designated *osu1-2*; F1, the progeny from the cross of *osu1-1* X *osu1-2*. (C) The two mutants, *osu1-1* and *osu1-2*, showed enhanced sensitivities to increasing concentrations of sucrose (0C to 210C) at 30N after 7 days of vertical growth. For 30C/30N+180 Man, 180 mM of mannitol was added to the medium containing 30 mM Suc, so that the total carbohydrate concentration of the medium was equivalent to 210 mM Suc. (D) Quantitative analysis of cotyledon anthocyanin accumulation in response to various Suc (C) concentrations at 30N. FW, fresh weight. (E) Quantitative analysis of cotyledon anothcyanin accumulation in response to the same concentration (210 mM) of Suc, glucose (Glc) and Mannitol (Man). No sugar supplementation was used as the control. (F) Quantitative analysis of cotyledon anothcyanin accumulation in response to various concentrations of maltose and 3-*O*-methylglucose (OMG). No sugar supplementation was used as the control.

During the process of screening for homozygous transgenic lines expressing a *ROP2-*promoter:*GUS* construct (*P_ROP2_:GUS*) in half-strength (1/2X) MS medium supplemented with 1% sucrose (Suc) and hygromycin, we found two T2 plants (23G13-2 and 23G9-8) from independent T1 transgenic lines that segregated for reddish purple and normally green cotyledons in the T3 generation. The progeny of seven T2 siblings did not (Figure 1A and data not shown). For convenience, the concentration of supplemental C and N will be expressed as milimolar concentrations in this study. A combination of 30 mM Suc and 30 mM of total N was designated 30C/30N. The 1% Suc (equivalent to 29 mM or 29C in this report) in 1/2X MS medium that contains 30 mM N (composed of 19.7 mM NO_3_^−^ and 10.3 mM NH_4_^+^ and designated 30N) did not induce the accumulation of sufficient anthocyanins to cause cotyledons to turn reddish purple in WT plants or the other transformants. This indicates that lineages 23G13-2 and 23G9-8 may carry mutations that led to the induction of anthocyanin production.

The progeny of 23G13-2 showed a clear 3∶1 segregation ratio (36∶14, χ^2^-value = 0.24, *p*-value >0.05) of green versus reddish purple cotyledons. F1 progeny after backcrossing to WT had normal green cotyledons, and their F2 progeny from the F1 selfcross also showed a clear 3∶1 segregation (157∶45, χ^2^-value = 0.80, *p*-value >0.05). T3 progeny from 23G9-8 showed a deviation from 3∶1 ratio (42∶5, χ^2^-value = 5.17, *p*-value<0.05). One of the plants showing anthocyanin accumulation, 23G9-8-1, was backcrossed to the Columbia (Col) WT and the resulting F1 showed normal cotyledons (data not shown). F2 plants exhibited a 3∶1 ratio of segregating green versus reddish purple cotyledons (57∶18, χ^2^-value = 0.04, *p*-value >0.05). These genetic analyses confirmed that these two lines carried monogenic, recessive mutations that caused the Petri-plate grown seedlings to accumulate more anthocyanins.

To determine whether the phenotype is genetically linked to the *P_ROP2_:GUS* construct, co-segregation analysis was performed using the F2 progeny from 23G13-2 crossed to WT. Results showed a segregation of 58 (green cotyledons and GUS positive): 16 (green cotyledon and GUS negative): 19 (reddish purple cotyledons and GUS positive): 4 (reddish purple cotyledons and GUS negative). This is consistent with a segregation ratio of 9∶3∶3∶1 (χ^2^-value = 1.22, *p*-value >0.05) for independent assortment of two loci. Therefore, the reddish purple cotyledon phenotype in 23G13-2 is unlinked to the T-DNA insertion. Mutations that occurred during T-DNA transformation but unrelated to the T-DNA insertion have been reported such as *abi5* [38].

F2 homozygous plants, selected for the absence of the *P_ROP2_:GUS* T-DNA insertion (determined by the absence of hygromycin resistance and GUS staining) from 23G13-2 and 23G9-8-1, were obtained. To determine whether 23G13-2 and 23G9-8-1 represent different loci or alleles of the same locus, they were crossed. The F1 progeny were indistinguishable from their parental plants when grown on the medium supplemented with 120 mM Suc (Figure 1B). Together, these results suggest that the Suc-enhanced phenotypes of 23G13-2 and 23G9-8-1 are due to mutant alleles at the same locus and therefore are designated *osu1-1* and *osu1-2*, respectively.

To further characterize the *osu1* Suc response phenotype, homozygous F3 *osu1-1* and *osu1-2* mutant plants were grown vertically on agar-solidified media supplemented with various Suc concentrations. Surprisingly, we found that at 30C/30N, cotyledons of 23G13-2 and 23G9-8-1 F3 seedlings did not appear reddish purple (Figure 1C) as was the case for horizontally grown seedlings on 1% Suc (29C/30N; Figure 1A). However, measurement of anthocyanin levels showed that they still had slightly higher anthocyanin levels than WT (Figure 1D). The smaller difference in anthocyanins between WT and the mutants under 30C/30N (Figure 1, C and D) than that under 29C/30N (Figure 1A) was due to vertical versus horizontal growth, instead of the presence or absence of hygromycin in the medium or the difference between 29C and 30C (data not shown). When C was increased at a constant N concentration from 30C/30N to 120C/30N and 210 C/30N, vertically grown WT seedlings showed increasing accumulation of anthocyanins. Both *osu1-1* and *osu1-2* mutants accumulated more anthocyanins than WT (Figure 1D). At 300C/30N the anthocyanin levels in WT were similar to that in both mutants (Figure 1D), indicating a possible saturation in the response. In the absence of supplemental Suc (0C/30N), both mutants had similar anthocyanin levels but shorter roots than WT plants (Figure 1C and D).

It has been shown that fructose and sorbitol do not induce anthocyanin accumulation, while maltose is similar to Suc, and glucose has an intermediate effect [39]. We tested the effects of using glucose and maltose as the source of C on anthocyanin accumluation. When glucose was used, both mutants also exhibited increases in anthocyanin accumulation, although *osu1-1* was less sensitive than *osu1-2* (Figure 1E). Similar to the results with Suc (Figure 1D), *osu1-1* and *osu1-2* accumulated more anthocyanins than WT on plates supplemented with maltose (Figure 1F). To exclude the possibility that the sugar effect is due to osmosis, we tested the seedling growth in the presence of mannitol and 3-*O*-methylglucose (a non-metabolizable glucose analog). The mutants were slightly less sensitive than WT to 3-*O*-methylglucose which weakly induced anthocyanin accumulation (Figure 1F and [40]). Growth on the medium supplemented with mannitol had a similar effect on anthocyanin accumulation (Figure 1E). When 180 mM mannitol was added to the medium supplemented with 30 mM Suc, we found that both of the mutants exhibited similar inhibition of root growth as WT (Figure 1C). Taken together, the two mutants greatly enhanced anthocyanin accumulation in response to high concentrations of metabolizable sugars (Suc, maltose and glucose) but not non-metabolizable or slowly metabolized sugars (3-*O*-methylglucose and mannitol). Thus, the enhanced sugar sensitivity of these mutants is not due to osmotic sensitivity.

### The *osu1* mutants are sensitive to an imbalance of C and N

The *osu1* mutant seedlings were hypersensitive to both high C/low N (120C/30N or 210C/30N) and low C/high N (0C/30N) but exhibited similar anthocyanin levels and root growth as WT plants when grown under more balanced C/N conditions (e.g. 30C/30N; Figure 1C). This led us to hypothesize that *osu1* mutants could be more sensitive to an imbalance of C and N. If this is the case, *osu1* seedlings should have similar anthocyanin levels and root lengths as WT when grown in medium with balanced C/N conditions regardless of absolute C or N concentration. Therefore, we formulated growth media with combinations of various C and N levels, and observed root growth and cotyledon anthocyanins in WT and *osu1* mutant plants [6].

**Figure 2:**
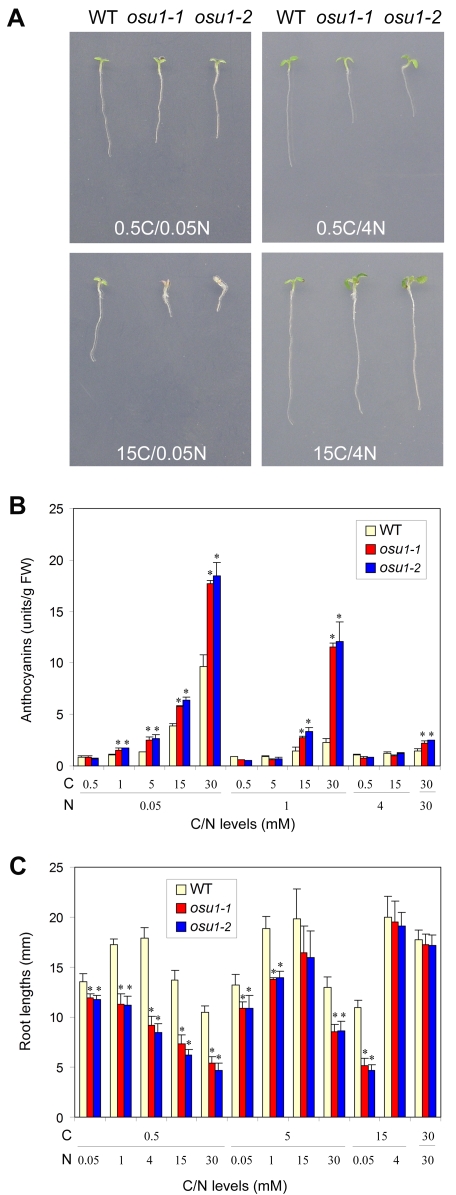
The *osu1* mutants increase the sensitivity to the imbalanced C/N. (A) Representative seedlings of WT, *osu1-1*, and *osu1-2* after seven days of growth on 0.5C/0.05N, 0.5C/4N, 15C/0.05N, and 15C/4N. (B) Quantitative analysis of cotyledon anthocyanin accumulation under various C/N conditions. Cotyledons excised from the 7-day-old seedlings were used to measure anthocyanin levels in relative units per g of fresh weight (FW). Shown are the average and the SD of three replicates, each with 8–10 seedlings. (C) Quantitative analysis of root growth inhibition under various C/N. Primary root lengths were measured. The average of four replicates (each with 8–10 seedlings) is shown, with the bar representing the SD. Statistical analysis by one way ANOVA; the asterisk (*) above the column indicates a significant difference (p<0.05) between *osu1-1* or *osu1-2* and wild-type (WT) under the same C/N condition.

When grown in medium with 0.5C/0.05N, *osu1-1* and *osu1-2* seedlings showed pale green cotyledons similar to WT. The roots of the *osu1* mutant seedlings were slightly (less than 2 mm or about 10%) shorter than WT (Figure 2, A and C). Quantitation of anthocyanin levels showed that the *osu1* mutants accumulated similar levels of anthocyanins in the cotyledons as WT (Figure 2B). However, when C increased from 0.5 to 1 or 5 mM while N was kept constant (0.05N), *osu1-1* and *osu1-2* accumulated more anthocyanins while WT did not (Figure 2B). As C levels increased further (15C/0.05N and 30C/0.05N), anthocyanin contents increased in WT cotyledons but *osu1* still had higher anthocyanin levels than WT. Similar results were obtained comparing medium with varying levels of C and 1N. The *osu1* mutants had higher anthocyanin levels when C increased to 15 or 30 mM. These observations are consistent with the earlier observation that *osu1* also had higher anthocyanin levels when C increased from 30 to 120 or 210 in the presence of 30N (Figure 1D). Consistent with the observation that the high C-induced anthocyanin accumulation is dependent on low levels of N [6], the anthocyanin levels increase when the N levels in the medium decrease from 1 to 0.05 mM while C is either 5C, 15C, or 30C (Figure 2B). Importantly, there was no difference between *osu1* and WT under 5C/1N, while decreasing N from 1 to 0.05 mM resulted in higher anthocyanin levels in *osu1*. Similarly, although *osu1* had no difference in anthocyanin levels under 15C/4N compared to WT, reducing N from 4 to 1 mM caused *osu1* mutants to accumulate more anthocyanins while WT did not. If N was further reduced from 1 to 0.05 mM in the presence of 15C, WT plants accumulated anthocyanins although *osu1* exhibited a similar increase as WT. In media with 30C, decreasing N from 30 to 1 mM resulted in a larger increase of anthocyanin levels for the *osu1* mutants than in WT. Further decreasing N to 0.05 mM caused a similar increase in WT and *osu1*. The *osu1* mutants increased anthocyanin accumulation in response to increased C when N was kept constant and to decreased N when C was kept constant. Thus, the accumulation of anthocyanins in *osu1* mutant seedlings was hypersensitive to the nutrient combinations with higher concentrations of C relative to N.

The enhanced sensitivity of *osu1* mutants to the low C/high N imbalanced conditions was also observed in the root growth assay. Increasing N from 0.05 to 30 mM in medium with 0.5C caused progressively shorter root lengths in the *osu1* mutants as compared to WT plants (Figure 2C). When N was varied across a higher (5C) concentration of Suc (Figure 2C) or no C was provided to the medium (Figure S1), *osu1* and WT exhibited similar responses to increasing N levels. To exclude the possibility that the observed root growth phenotype in *osu1* might be due to seed germination variation between *osu1* and WT, seed germination was recorded every two hours. The *osu1* mutants had very similar germination profiles as WT under four tested C/N conditions, 0.5C/0.05N, 0.5C/4N, 15C/0.05N, and 15C/4N (Figure S2). In addition, *osu1* differed from WT in both anthocyanin accumulation and root growth inhibition at 15C/0.05N but only the root growth inhibition at 0.5C/4N. This indicates that anthocyanin accumulation and root growth are likely two separable responses under the imbalanced C/N conditions.

### The *osu1* mutants do not increase anthocyanin accumulation in response to phosphate or sulphate nutrient deficiencies

**Figure 3:**
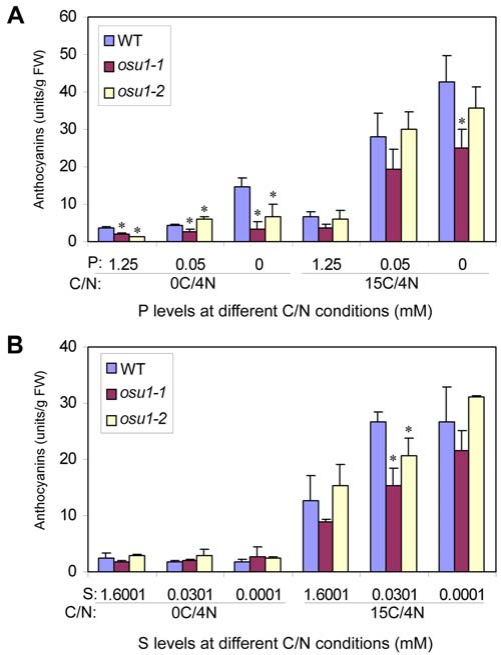
*osu1* does not increase the sensitivity to phosphate and sulphate nutrient deficiency-activated anthocyanin accumulation. (A) Anthocyanin accumulation under phosphate deficiency. Seeds were germinated and grown vertically on two C/N (0C/4N or 15C/4N) conditions with various phosphate (P) concentrations (0, 0.05, and 1.25 mM) for seven days. The average of three replicates (each with 6–8 seedlings) is shown, with the bar representing the SD. (B) Anthocyanin accumulation under sulphate deficiency. Seven-day-old seedlings vertically grown on the 15C/4N plates were transferred to liquid 0C/4N or 15C/4N medium supplemented with various sulphate (S) concentrations (0.0001, 0.0301, and 1.6001 mM) for additional three days. The average of three replicates (each with 5 or 6 seedlings) is shown, with the bar representing the SD. FW, fresh weight. Statistical analysis by one way ANOVA; the asterisk (*) above the column indicates a significant difference (p<0.05) between *osu1-1* or *osu1-2* and wild-type (WT) under the same C/N/P or C/N/S condition.

Anthocyanin accumulation is also activated by phosphate or sulphate nutrient deficiencies, which are also dependent on C availability [40]–[42]. We tested whether *osu1* mutants were more sensitive than WT to phosphate or sulphate deficiencies in the presence or absence of 15C. Comparison of cotyledon anthocyanin levels in 0C/4N and 15C/4N-treated 7-day-old seedlings grown vertically in the presence of various phosphate levels showed that C greatly promoted phosphate deficiency-activated anthocyanin accumulation (Figure 3A). At 0C/4N, WT plants accumulated more anthocyanins in the absence than the presence of phosphate (Figure 3A). Anthocyanin accumulation was not sufficient to cause cotyledons to turn visibly reddish purple (data not shown). Nevertheless, *osu1* did not increase the sensitivity to phosphate deficiency and indeed had slightly lower anthocyanin levels than in WT in most of the conditions. In medium containing 15C/4N, both *osu1* and WT had similar activation of anthocyanin accumulation in response to phosphate deficiencies except that *osu1-1* had slightly lower anthocyanin levels than WT (Figure 3A).

Seedlings grown vertically on agar-solidified medium with a deficiency of sulphate were not visibly red (data not shown). Seedlings in liquid culture under sulphate deficiency (0.0001 mM) accumulated one-fold higher anthocyanin levels than the sulphate sufficiency (1.6001 mM) control in a previous study [40]. We grew seedlings on the medium containing 15C/4N with a sufficient sulphate supply for seven days and then transferred them to liquid media supplemented with three different sulphate levels and both 0C/4N and 15C/4N conditions. Sulphate deficiency activated anthocyanin accumulation, dependent on the availability of C (Figure 3B). This effect was greater than that observed during phosphate deficiency response (Figure 3A). Under 0C/4N, sulphate deficiency did not activate anthocyanin accumulation. The *osu1-1* and *osu1-2* mutant plants exhibited very similar patterns of anthocyanin accumulation as WT in response to sulphate deficiency. The only difference was a slightly lower anthocyanin level in *osu1* than the WT at 0.0301 mM sulphate under 15C/4N (Figure 3B). Phosphate or sulphate nutrient deficiency activated anthocyanin accumulation but *osu1* mutants were not enhanced in these responses. This suggests that OSU1 functions in the response to C and N nutrient balance but is not involved in a response shared by nutrient deficiencies in general.

### *OSU1* suppresses expression of *MYB75* in high C/low N

**Figure 4:**
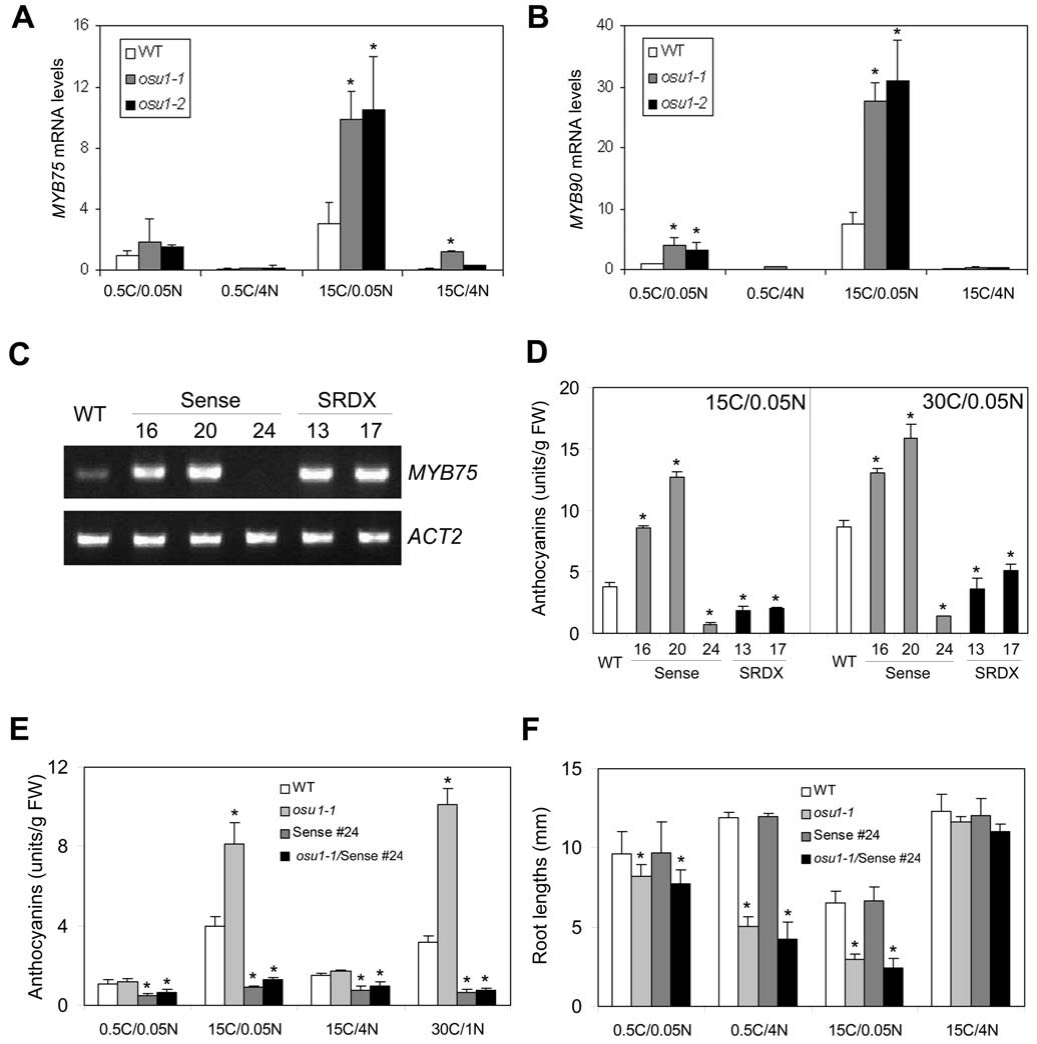
OSU1 acts upstream of MYB75 in response to high C/low N. (A and B) The *OSU1* mutations altered *MYB75* and *MYB90* expression in response to high C/low N. Four-day-old WT, *osu1-1* and *osu1-2* seedlings grown vertically on 15C/4N were transferred to freshly prepared media with four different C/N conditions for three days of treatment. *MYB75* (A) and *MYB90* (B) mRNA levels were normalized with those of the reference (*ACT2*), and the relative *MYB75* or *MYB90* mRNA level under 0.5C/0.05N was set at 1. The average and the SD bar for *MYB75* (n = 4) and *MYB90* (n = 3) are shown. (C) RT-PCR analysis of *MYB75* expression in *MYB75* sense (#16, 20 and 24) and dominant negative SRDX lines (#13 and 17). Primers were used to amplify both endogenous and transgenic *MYB75* expression. *ACT2*, an internal control. (D) Anthocyanin accumulation of the transgenic lines under high C/low N. The averages and SDs of three replicates, each with 10-15 seedlings, are shown. FW, fresh weight. (E and F) The *MYB75* co-suppression line (Sense #24) is epistatic to *osu1-1* at high C/low N. (E) Anthocyanin levels, and (F) root lengths at four different C/N conditions, each with four replicates. *osu1-1*/Sense #24 represents a homozygous line of *osu-1* and *MYB75* co-suppression. Statistical analysis by one way ANOVA; the asterisk (*) above the column indicates a significant difference (p<0.05) from wild-type (WT) under the same C/N condition.

We investigated whether the *osu1* alleles affect the transcript levels of two MYB transcription factor genes, *MYB75/PAP1* and *MYB90/PAP2*, which are involved in activating anthocyanin biosynthesis in response to C and N nutrient status [39], [43]–[46]. Real-time RT-PCR analysis showed that in medium with 15C/0.05N, both *osu1-1* and *osu1-2* had greater *MYB75* expression as compared to WT. Only *osu1-1* had higher *MYB75* expression levels than WT under 15C/4N, while *osu1-2* was similar to WT (Figure 4A). At 0.5C/4N, both mutants and WT had similarly low levels of *MYB75* expression. This pattern of transcriptional regulation is consistent with the phenotypic observation that *osu1* had similar anthocyanin levels as WT under 0.5C/4N (Figure 2B). A similar response pattern for *MYB90* was observed except that *osu1-1* and *osu1-2* had slightly higher *MYB90* transcript levels under 0.5C/0.05N (Figure 4B). These results indicate that *osu1-1* and *osu1-2* up-regulates *MYB75* and *MYB90* induction by high C/low N.

To confirm that *OSU1* acts upstream of *MYB75* and to test the hypothesis that anthocyanin accumulation and root growth are two separable responses under the imbalanced C/N conditions, an epistasis study was performed. We generated transgenic plants in the Col background that express a dominant negative form of MYB75 by fusing MYB75 to the SUPERMAN repression domain (SRDX) which has been shown to suppress the function of several transcription factors [47], under the control of the constitutively active UBQ10 promoter [48]. We obtained two lines that overexpressed *MYB75-SRDX* (lines #13 and 17; Figure 4C) which showed the expected green cotyledon phenotypes under 30C/1N (data not shown) due to reduced anthocyanin accumulation as compared to WT (Figure 4D). Stronger knock-down of *MYB75* was obtained from co-suppression of the endogenous *MYB75* by a construct designed to overexpress *MYB75* cDNA. RT-PCR analysis showed that while two overexpression lines (#16 and 20) accumulated more *MYB75* transcripts than WT, the co-suppressed line (#24) accumulated far less *MYB75* mRNA (Figure 4C). Real-time PCR analysis showed that the total transcript level of both endogenous and transgenic *MYB75* in Sense #24 was reduced to only 9% of *MYB75* levels in WT. Consistent with their *MYB75* expression levels, the overexpressors (Sense #16 and #20) had more anthocyanins, while the *MYB75* co-suppression line (Sense #24) had less anthocyanin accumulation than WT when grown at 15C/0.05N (Figure 4D). This co-suppression line exhibited even stronger suppression of anthocyanin accumulation than the two SRDX lines under 15C/0.05N. Furthermore, in contrast to the two SRDX lines, Sense #24 only exhibited a very mild increase of anthocyanins when C increased from 15 to 30 mM (Figure 4D). Therefore, Sense #24 was used for the subsequent study.

Sense #24 was crossed to *osu1-1*, and the F3 seedlings from a homozygous *osu1-1*/Sense #24 plant were then subjected to various C/N treatments. When media containing 0.5C/0.05N and 15C/4N were used, there was no difference between WT and *osu1-1*, but Sense #24 had a slightly lower level of anthocyanins than WT (Figure 4E). Under these conditions, *osu1-1*/Sense #24 had similar anthocyanin levels as Sense #24 alone, indicating that *MYB75* co-suppression can block the production of anthocyanins in both WT and *osu1-1*. At 15C/0.05N, while *osu1-1* dramatically increased the anthocyanin levels, *osu1-1*/Sense #24 showed only a very small increase in anthocyanin accumulation. At 30C/1N, Sense #24 completely eliminated the activation of anthocyanin accumulation in *osu1-1* (Figure 4E). However, root growth inhibition in *osu1-1* was not restored by Sense #24 (Figure 4F). Neither did the co-suppression of *MYB75* alone affect root growth compared to WT. These results show that MYB75 is downstream of OSU1 in modulating anthocyanin induction and that anthocyanin accumulation and root growth in *osu1* mutants are two separable responses.

### *OSU1* suppresses *ASN1* expression in response to low C/high N

**Figure 5:**
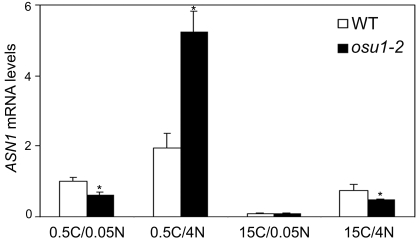
OSU1 suppresses *ASN1* expression in response to low C/high N. Seedlings were treated with various C/N conditions and real-time RT-PCR analysis was performed as described in Figure 4 legend. mRNA levels were normalized with those of the *ACT2* reference, and the relative *ASN1* mRNA level under 0.5C/0.05N was set at 1. The bar represents the SD of three replicates. Statistical analysis by *t*-test; the asterisk (*) above the *osu1-2* column indicates a significant difference (p<0.05) from wild-type (WT) under the same C/N condition.

The observation that *MYB75* and *MYB90* were affected by the *osu1-2* and *osu1-2* mutations under high C/low N led us to test whether the expression of an isoform of Asn synthetase (*ASN1)*, was affected by *OSU1*. *ASN1* mRNA expression has been shown to be regulated by light/sugar and amino acids and to correlate with amino acid biosynthesis [21], [22], [27]. *ASN1* expression under 0.5C/0.05N was 40% lower in *osu1-2* than WT (Figure 5). Increasing the N to 0.5C/4N caused an almost eight-fold increase of *ASN1* transcript levels in *osu1-2* compared to *osu1-2* at 0.5C/0.05N. This resulted in *osu1-2* mutants accumulating four fold more *ASN1* transcript than WT. *ASN1* expression was very low and similar in WT and *osu1-2* at 15C/4N. The *osu1-2* mutant was slightly reduced as compared to WT at 15C/4N, similar to what was found at 0.5C/0.05N. These results showed that the *OSU1* mutation increased the quantity of *ASN1* up-regulation by low C/high N, while the differences between WT and *osu1-2* under both of the balanced conditions (0.5C/0.05N and 15C/4N) were similar and small. This effect of *osu1-2* on *ASN1* expression under low C/high N is in sharp contrast to that for *MYB75* and *MYB90*, which were modulated by loss of OSU1 function under high C/low N growth conditions (Figures 4A and 4B).

### *OSU1* encodes a member of a large family of putative methyltransferases

**Figure 6:**
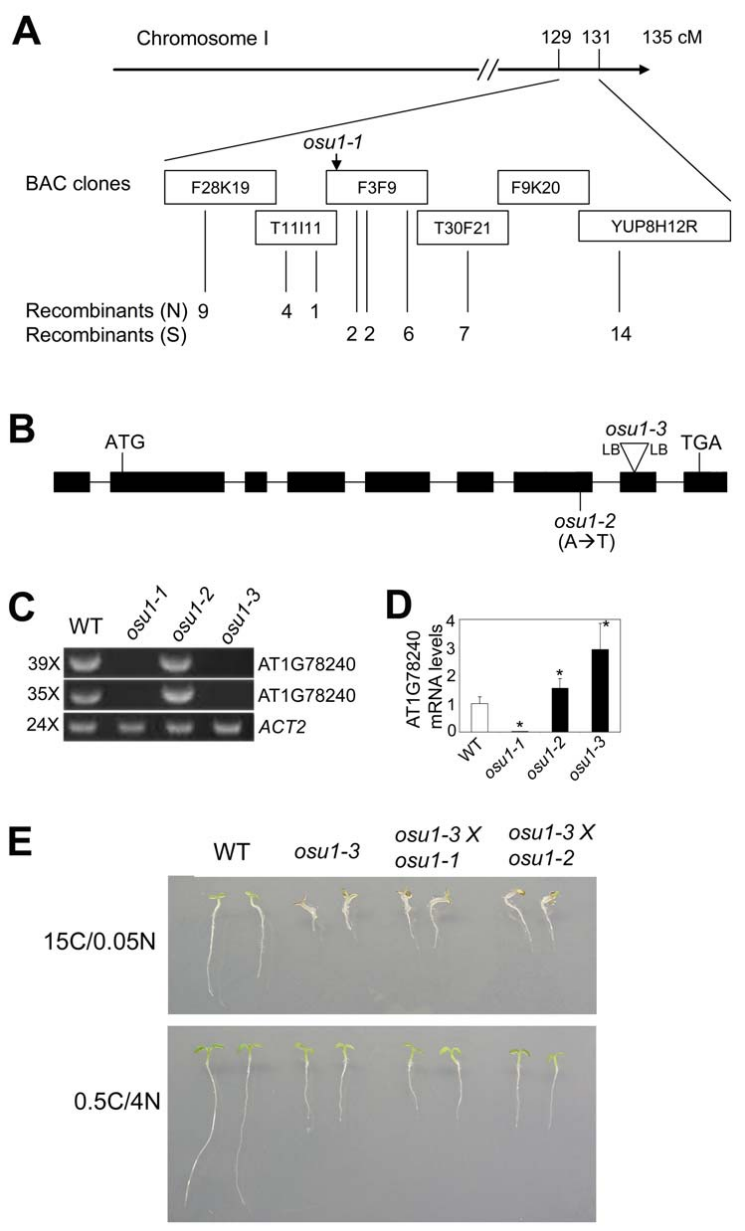
Molecular cloning of the *OSU1* gene. (A) A diagram showing the *osu1* mutant gene mapping. The number of recombinants (chromosomes) is indicated for each marker used on the BAC clones. The name and the primers for each marker are listed in Table S1. The *osu1-1* mutant gene is mapped to a region between two markers (470560 on the BAC clone T11I11, and (470251 on the BAC clone F3F9) that respectively gave one and two recombinants. (B) A schematic representation of the *OSU1* genomic structure. *osu1-3* had an insertion of T-DNA surrounded by its two left border (LB) sequences that eliminated 10 bp starting from the 54^th^ bp of the eighth exon (sequencing data for the T-DNA and genomic DNA junction not shown). The positions of start codon (ATG) and stop codon (TGA) were indicated. (C) Regular RT-PCR analysis of At1g78240 mRNA expression in the three *osu1* alleles. PCR reactions with 35 or 39 cycles were used to amplify the 2,055 bp CDS region of At1g78240. *ACT2* was used as an internal control. (D) Real-time PCR analysis of At1g78240 transcript levels. A 98 bp fragment of At1g78240 cDNA located in the second exon was amplified. Statistical analysis by one way ANOVA; the asterisk (*) above the column indicates a significant difference (p<0.05) from wild-type (WT). The bar represents the SD of three replicates. (E) *osu1-3* fails to complement *osu1-1* and *osu1-2*, respectively. The F1 seeds of the crosses were sown on the medium containing 15C/0.05N and 0.5C/4N, respectively, and two representative seedlings vertically grown for 7 days for each genotype are shown.

We cloned the *OSU1* gene using a map-based strategy [49]. The *osu1-1* mutation was mapped to a 35 kb region between two markers on the two BAC clones T11I11 and F3F9 (Figure 6A, and Table S1). The sequences of open reading frames for seven of the nine genes within this region had DNA sequences identical to the published genome sequence. The remaining two genes, At1g78230 and At1g78240, could not be PCR amplified from *osu1-1*. Sequencing of these genes from *osu1-2* revealed that At1g78230 was identical to the reference sequence, and an A to T mutation in the seventh exon of At1g78240 (Figure 6B) resulting in a missense mutation that converts Asn at 560 to Tyr (N560Y). The genomic lesion in the region of the At1g78240 gene was not characterized further in the *osu1-1* mutant, but it is likely that a DNA rearrangement and/or deletion is responsible for the mutation (Figure 6C and data not shown). To further support this, we isolated homozygous T-DNA insertion lines collected from The Nottingham Arabidopsis Stock Centre (GABI_Kat 107A03) and Arabidopsis Biological Resources Center (Salk_122037 and Salk_062502). RT-PCR analysis showed that the two Salk lines, which have T-DNA insertions in the promoter region, did not affect At1g78240 gene expression (data not shown). However, the 2,055 bp CDS (from ATG to TGA) of the At1g78240 full-length cDNA could not be amplified in the GABI_Kat 107A03 line (Figure 6C) due to a T-DNA inserted into the eighth exon of At1g78240 (Figure 6B). This GABI line was designated *osu1-3*. Consistent with a deletion in *osu1-1*, no transcript was detected in RT-PCR reactions from RNA isolated from homozygous mutant plants (Figure 6C). In contrast, At1g78240 expression was not suppressed in *osu1-2*, indicating that the phenotype is likely due to the N560Y mutation inactivating the OSU1 protein. To quantify the expression level of At1g78240 in these alleles using real-time PCR, two primers were designed to amplify a 98 bp fragment within the second exon 5′ of the *osu1-2* mutation and the *osu1-3* T-DNA insertion sites (Figure 6B). Using these primers, At1g78240 transcript levels in *osu1-2* and *osu1-3* were respectively 50% and two-fold higher than that in WT (Figure 6D). Surprisingly, these reactions also detected the At1g78240 transcript in *osu1-1*, although only 2% of that in WT. These results indicate that At1g78240 mRNA expression could be regulated by negative feedback. We found that *osu1-3* showed similar C and N responses as *osu1-1* and *osu1-2* (data not shown). Furthermore, *osu1-3* failed to complement *osu1-1* and *osu1-2* (Figure 6E). These results show that the *OSU1* locus is At1g78240.

The *OSU1* gene is predicted to contain nine exons and encodes a putative methyltransferase with 684 amino acids. In the *Arabidopsis* genome, there are a total of 28 additional putative methyltransferases that are closely related to OSU1 (Figure S3). Among these, one, At4g19120/*Early Response to Drought3* (*ERD3*), has been reported to exhibit mild transcriptional induction to drought [50]. The gene tree analysis, using IAMT1 [51] as an outgroup, indicates that the OSU1–related putative methyltransferases can be divided into six groups, I to VI (Figure S3). Group I has only one member encoded by At3g56080, while Group II contains OSU1 and two other members encoded by At1g13860 and At2g03480. Groups III-VI can be considered a sister to Group II. ERD3 belongs to Group V which is the largest group with 10 members. Amino acid sequence alignment shows that the *osu1-2* mutation affects a conserved amino acid N at 560 which is present within a highly conserved seven amino acid-containing motif, WVMNVVP. Only At2g39750 of Group IV (Figure S3), which has a serine residue at this position, lacks N_560_. Together with the fact that *osu1-2* (N560Y) does not reduce its transcript level (Figure 6C), this indicates that N_560_ is critical for the proper function of the *OSU1*-encoded protein.

### Expression patterns of *OSU1*

**Figure 7:**
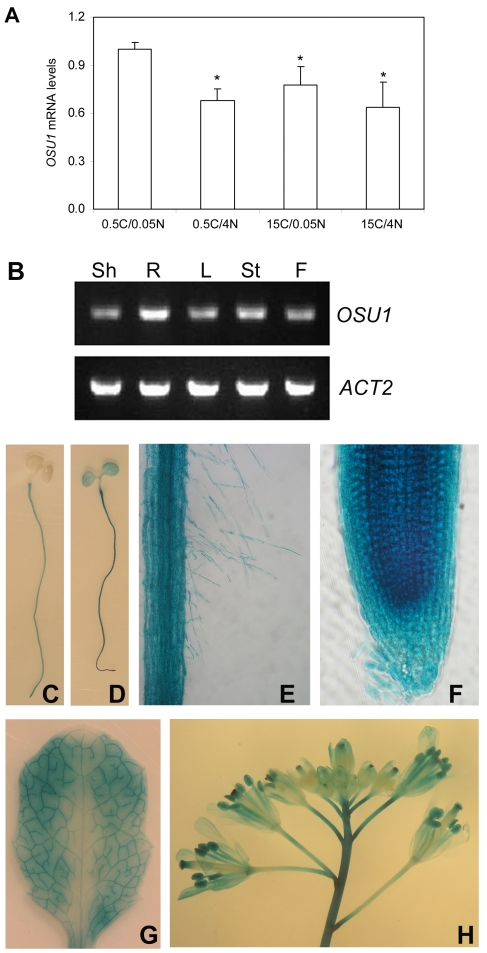
Expression patterns of *OSU1*. (A) Quantitative RT-PCR analysis of *OSU1* under various C/N conditions. WT seedlings were first grown vertically on agar-solidified 15C/4N medium for four days of optimal growth and then transferred to various C/N media for three days. mRNA levels were normalized with those of the *ACT2* reference, and the relative *OSU1* mRNA level under 0.5C/0.05N was set at 1. The bar represents the SD of three replicates. Statistical analysis by one way ANOVA; the asterisk (*) above the column indicates a significant difference (p<0.05) compared to the control (0.5C/0.05N). (B) RT-PCR analysis of *OSU1* expression in different organs. Shown are shoots (Sh) and roots (R) of 7-day-old seedlings, mature leaves (L), stems (St) and flowers (F). *ACT2*, an internal control. (C-H) Expression patterns revealed by histochemical examination of the *OSU1* promoter activity using the *GUS* reporter. Shown are a 7-day-old young seedling stained for 4 hours (C) and another for 10 hours (D), enlarged views of root hairs (E) and a root tip (F), a mature leaf (G), and an inflorescence with several flowers (H).

We tested whether C and N nutrient imbalance affects *OSU1* transcription. Real-time RT-PCR analysis of *OSU1* transcript levels at 0.5C/4N, 15C/0.05N and 15C/4N were quite similar (Figure 7A). The former two nutrient regimes displayed increased root growth inhibition or anthocyanin accumulation in *osu1* as compared to WT controls (Figure 2). WT plants under these conditions accumulated 20–40% lower levels of *OSU1* transcript than plants at 0.5C/0.05N (Figure 7A), which did not induce anthocyanins in either *osu1* mutants or WT. Thus *OSU1* mNRA accumulation is regulated by C and N nutrient status, but only to a limited degree. To reveal organ-specific regulation, we performed RT-PCR analyses using RNAs extracted from young seedlings (roots and shoots), mature leaves, stems and flowers (Figure 7B). *OSU1* was expressed in all of these organs, with higher expression in the roots than the shoots of young seedlings. Transgenic plants were generated containing 3.2 kb of *OSU1* genomic DNA that includes the first exon, the first intron, and part of the second exon (up to 24 bp downstream of the ATG start codon) fused in frame to *β-glucuronidase (GUS)*. GUS staining of multiple independent transgenic lines showed that the *OSU1* promoter was more active in the root than in the shoot (Figure 7, C and D), consistent with the RT-PCR result (Figure 7B). Weak GUS staining was detected in root hairs (Figure 7E) and all cell layers of the root tip (Figure 7F). Staining was slightly stronger in the vascular tissues of the root (Figure 7, E and F) and in the vascular tissues of mature leaves (Figure 7G). In inflorescence, the promoter is active in stems, anthers, and stigma surface (Figure 7H).

## Discussion

Despite the fact that C and N influence the expression of many genes [7], [8], [20], [24], the genetic control of the response to the imbalanced C/N conditions remains to be determined. It has been shown that the *lin1* mutant, which has a mutation in a nitrate transporter (NRT2.1) gene, reduces the sensitivity to high C/low N, but it is unknown whether *lin1* alters the response to low C/high N [3], [29]. Here, we report that the *osu1* mutants increase the sensitivity to both high C/low N and low C/high N, suggesting that the *OSU1*-encoded putative methyltransferase acts as a negative modulator of the C/N nutrient imbalance response. *osu1-1* and *osu1-2* were initially isolated as sugar oversensitive mutants, but their response to sugars depends in part on the C/N ratio rather than responding solely to C. It should be noted that the *osu1* mutants did not show consistent hypersensitivity to the same C∶N ratios when different absolute levels of C or N were compared. For example, under 15C/4N (a C∶N ratio of close to 4), *osu1* and WT seedlings had similar root lengths and anthocyanin levels (Figure 2), but 120C/30N ( a ratio of 4) led *osu1* mutants to accumulate more anthocyanins than WT (Figure 1D). More importantly, under intermediate C/intermediate N (15C/4N) and high C/high N (30C/30N), the *osu1* mutants and WT had no or only a marginal difference in both anthocyanin accumulation and root growth. Phenotypes of previously described mutants or transgenic plants that alter C or N responses should be re-examined under a wider spectrum of C and N levels (Figure 2). Some of the mutants or transgenic plants include genes in regulatory pathways such as the hexokinase pathway [52]–[54], heterotrimeric G-proteins [55], and the ANR1 transcription factor [56].

The response to C/N ratio in *osu1* mutants suggests that OSU1/QUA2/TSD2 could be involved in nutrient utilization or signaling. The utilization of C (such as glycolysis or citric acid cycle) is controlled by N availability and similarly the utilization of N (such as nitrate or ammonium assimilation) is modulated by C availability [1], [2]. Therefore, OSU1 might have a strong effect on overall metabolism and affect the response by altering the concentration of substrates critical in the C or N sensing pathways. Proteins involved in C or N transport and metabolism such as HXK1/HXK2, TPS1 and LIN1 also have a regulatory role in C or N signaling [3], [29], [53], [54], [57]. Clearly, it is necessary to dissect the biochemical mechanism by which loss of OSU1 function enhances the response to changes in the ratio of C to N.

Several of our results suggest that the enzyme activity of OSU1/QUA2/TSD2 is necessary for its function in C and N balance signaling. First, the accumulation of *OSU1/QUA2/TSD2* transcript was not dramatically altered by changes to C and N conditions (Figure 7A). Second, the single amino acid replacement (N560Y) in *osu1-2* does not lead to its transcriptional suppression but has a strong phenotype (Figures 2 and 6). This mutation in *osu1-2* occurs within a conserved seven amino acid motif in the predicted methyltransferase domain (Figure S3). AdoMet-dependent methyltransferases add a methyl group to various biologically active molecules such as proteins, lipids, DNA/RNA, and secondary products. Although they share little sequence identity, around 120 members have been classified (EC 2.1.1.X), based on the substrate specificity and on the atom targeted for methylation [58]. The observations that *osu1/qua2/tsd2* mutants exhibit defects in cell wall composition or structure and hypersensitivity to C/N balance indicate a possible link between cell wall modifications and C/N nutrient balance response. The *qua2* mutant exhibits a 50% reduction in homogalacturonan content compared to WT [37]. Our observed C/N response phenotypes could be due to these cell wall defects. Alternatively, loss of OSU1 function could result in the production of oligosaccharides with signaling activity, or a disruption in *S*-adenosylmethionine utilization, either of which might affect the C/N signaling mechanism. The hypothesized role of QUA2/TSD2 as a pectin methyltransferase has not been demonstrated. We failed to express and purify OSU1 in bacteria using various protein tags (data not shown), and the difficulty was also mentioned in another report [37]. Therefore, it remains unclear at this time whether OSU1-modulated cell wall modification is direct and how it might be involved in the C/N balance response including anthocyanin biosynthesis and root growth. Dissection of the OSU1 biochemical and regulatory functions will help elucidate the mechanism by which plants, and possibly other cellular organisms, respond to C and N nutrient balance.

## Materials and Methods

### Plant growth and materials

*Arabidopsis thaliana* Columbia (Col) wild-type, transgenic plants and mutants in the Col background were used in this study. Seeds were cold-treated at 4°C for 2–4 days and then allowed for germination and growth in the greenhouse or incubator at 22°C with 16 h light and 8 h dark.

### Isolation of the *osu1* mutants and cloning of the *OSU1* mutant gene

The *osu1* mutants were isolated from T3 seedlings that carried the *P_ROP2_:GUS* construct (see below) and grown in the half-strength (1/2X) MS medium supplemented with 1% sucrose and 50 µg/mL hygromycin. The mutants were then backcrossed to Col to remove the T-DNA transgene background. For map-based cloning, *osu1-1* was out-crossed to the ecotype L*er*, and the F2 seedlings from the F1 self-cross were selected for the *osu1* mutant phenotype. Genomic DNAs from a total of 544 F2 plants showing the *osu1* phenotype were extracted for marker genotyping. Simple Sequence Length Polymorphism (SSLP) markers representing distinct regions of each chromosome were first used for rough mapping according to Lukowitz et al. [49]. After we showed that the *OSU1* mutant gene was mapped to the bottom arm (or the South end) of chromosome 1 between the markers, NGA111 and F23A5, fine mapping was performed by designing a set of primers (Table S1). As shown in Figure 6A and Table S1, the *OSU1* mutant gene was eventually mapped to a region of 35 kb. Subsequently, various fragments of the open reading frames for the nine genes included in this region were PCR-amplified using the primers listed in Table S1 and then sequenced to determine where the mutation was located. The *osu1-2* allele was also used for DNA amplification in order to verify the mutant gene was represented by At1g78240. The *osu1-3* allele (GABI_Kat 107A03 in which a T-DNA insertion was verified by sequencing the junction between its left border and the *OSU1* eighth exon) was isolated from a collection maintained in The Nottingham Arabidopsis Stock Centre. For screening the T-DNA insertional plants, two primers were used to determine the insertion of T-DNA: GZP61 (Table S1) and GAB8409L (5′-ATATTGACCATCATACTCATTGC-3′), according to Rosso et al. [59]. GZP61 and GZP62 (Table S1) were used to screen homozygous plants for the T-DNA insertion. Subsequently, the genomic region for the other side of T-DNA and *OSU1* junction was sequence verified using the PCR fragment amplified with the primers GZP62 and GAB8409L.

### C and N balance response assays

The assays were modified from Martin et al. [6] and the various C/N media were prepared according to Dan et al. [40]. In brief, 1/2XMS salts without KNO_3_, NH_4_NO_3 _and sucrose was prepared at pH value of 5.7, and then various C and N were added to the media. Sucrose was used as the C source, and the combination of NO_3_^−^ and NH_4_^+^ in a molar ratio of 2∶1 was used as the N source. All the media contained similar amount of K^+^ by replacing KNO_3_ with KCl, if necessary. 1% PhytoBlend (Caisson Laboratories Inc., USA) was added to solidify the medium. After 2–4 days of cold treatment, seeds were allowed to germinate and grow vertically for seven days. Anthocyanin contents were measured using the methanol-based extraction (99% methanol plus 1% concentrated HCl) method as described [40]. Lengths of the primary roots of the seedlings were measured using a ruler. RNA for RT-PCR was prepared from seedlings treated as follows: seeds were germinated and grown vertically under the balanced C/N (15C/4N) for four days and then transferred to the agar-solidified media of various C/N conditions for three more days of vertical growth.

### Phosphate and sulphate deficiency response assay

For phosphate deficiency, seeds were sown and seedlings were grown vertically in the plates containing various C and phosphate levels for seven days. Phosphate deficiency media were prepared by adding KH_2_PO_4_ at various levels to 0C/4N and 15C/4N, adjusted by adding KCl to keep the [K^+^] constant. For sulphate deficiency, seven-day-old seedlings grown vertically under 15C/4N with sufficient sulphate were transferred to the 0C/4N and 15C/4N liquid media supplemented with various sulphate levels prepared as described elsewhere [40]. Anthocyanin contents were measured as described above.

### Production of *P_ROP2_:GUS* and *P_osu1_:GUS* transgenic plants

For the *P_ROP2_:GUS* construct, a 2.3 kb *ROP2* promoter fragment ending 2 bp upstream of ATG was PCR amplified from genomic DNA using the high fidelity DNA polymerase PfuUltra™ (Stratagene, USA) and the following gene-specific primers, with the underlined bases indicating the introduced restriction enzyme sites for cloning: sense (ZZP1: 5′-ATCTGCAGTTGCCTTCCTTCCTATGTACGTA-3′) and antisense (ZZP3: 5′-CGATCCATGGCTGCCGCAAGATCGGAAACAA-3′). The amplified DNA fragment was digested by *Pst* I and *Nco* I, and then cloned into *Pst* I and *Nco* I sites of the binary vector pCAMBIA1301 (B4) that contains GUS and the CaMV 35S terminator. This resulted in the plasmid, ZZ102. For the *P_osu1_:GUS* construct, a 3.2 kb promoter fragment, including the first exon, the first intron and part of the second exon (up to 24 bp downstream of ATG), was similarly PCR amplified using the gene-specific primers, with the underlying bases indicating the introduced restriction enzyme sites for cloning: sense (GZP69: 5′- CATCTGCAGGTCACGGTCCGCCACACACAAGA-3′) and antisense (GZP71: 5′- CCTCTAGAGCCACGTTGTAGTGGCATTGACAT-3′). The amplified DNA fragment was digested with *Xba* I, and then cloned into the *Hind* III (fill-in) and *Xba* I sites of the binary vector pBI101 that contains *GUS* and the *NOS* terminator. This resulted in the plasmid, GZ59. ZZ102 and GZ59 were then transformed into Col using the floral-dip method [60]. Multiple independently transformed lines were used for GUS activity assay as described elsewhere [61].

### RT-PCR analysis

Total RNA was extracted with TRIzol (Invitrogen, USA) and 2–6 µg RNA were reverse transcribed in a 20-µL reaction using Superscript III reverse transcriptase and Oligo(dT)_12–18_ primer (Invitrogen), according to the instructions provided by the vender. Regular PCR analysis was performed using Taq DNA polymerase (GenScript, USA), with *ACT2* as the internal control using the primers ACT2S and ACT2A described elsewhere [62]. Gene specific primers for *OSU1* were: ZZP87 (sense) 5′- CAGGATCC ATGTCAATGCCACTACAACGTG -3′ and ZZP88 (antisense) 5′- GTCTGCAG TCAGATTGATTGTCGCTTGGTG -3′. For *MYB75*, they were: GZP7 (sense) 5′-TTCCATGGAGGGTTCGTCCAAAGGGCTGC-3′, and GZP8 (antisense) 5′-GCTTCAGGAACCAAAATATCTACC-3′. Key parameters of PCR were provided in Table S2.

For real-time quantitative PCR analysis, the QuantiTect SYBR Green PCR kit (Qiagen, USA) was used according to the instructions provided by the vender. Real-time PCR was carried out in the MasterCycler II (Cypheid, USA) according to the manufacturer's protocol, with key parameters of PCR provided in Table S2. The primers were designed using the software provided online by GenScript (http://www.genscript.com/bioinformatics.html), and the *ACT2* primers (ActSDS and ActSDA) were designed previously [63]. Gene-specific primers for *OSU1* were: ZZP89 (sense) 5′- CTTGCTGGTTCTTTCTGGTG-3′ and ZZP90 (antisense) 5′- TCTGAAACAAGCTGCTCCTG-3′. Primers for *MYB75* were: GZP91 (sense) 5′-GACTGCAACCATCTCAATGC-3′) and GZP92 (antisense) 5′-TTGGTCTTTCTTCTTATCTTTGTTG-3′). Primers for *MYB90* were: ZZP97 (sense) 5′- CAAGAAGCTGATGCGATTGT-3′ and ZZP98 (antisense) 5′- AACGTCAAACGCCAAAGTG-3′. Primers for *ASN1* were: ZZP83 (sense) 5′- ACTCTTTCATGGTGGCTCGT-3′ and ZZP84 (antisense) 5′- ACGTTTCGAAATGCTCACAG-3′.

### Production of *MYB75* transgenic plants

The vector with the *UBQ10* promoter, designated ZZ106, was first constructed by replacing the CaMV 35S promoter of the vector pCAMBIA1301 (B4) with the PCR-amplified 1 kb promoter fragment of the *UBQ10* (At4g05320) gene [48], using the primers ZZP6 (sense, 5′-TACTGCAGACGGATCAGGATATTCTTGT-3′) and ZZP7 (antisense, 5′-CGATCCATGGCGGTAGAGAGAATTGAGAGA-3′) with the restriction sites underlined. For *MYB75*-overexpression and MYB75-SRDX constructs, the SRDX fragment containing twelve amino acids LDLDLELRLGFA encoded by part of the *SUP* gene as described in [47] was first amplified with the primers ZZP48 (sense, 5′-GTAGATCTAGGT ACCCTCGATCTGGATCTAGAACTCCGT-3′) and ZZP49 (antisense, 5′-GTGTTAAGCGAAACCCAAACGGAGTTC-3′) and then cloned into GZ10, resulting in the vector GZ36. The *UBQ10* promoter fragment was digested with *Pst* I (fill-in) and *Nco* I from ZZ106 and then cloned into GZ36, resulting in the vector GZ41. *MYB75* cDNA was then PCR amplified using the primers GZP7 (sense, 5′-TTCCATGGAGGGTTCGTCCAAAGGGCTGC-3′) and GZP9 (antisense for MYB75-SRDX, 5′-ATGGTACCATCAAATTTCACAGTCTCTCCATCG-3′ that does not include the stop codon) and GZP10 (antisense for *MYB75*-overexpression, 5′- ATGGTACCCTAATCAAATTTCACAGTCTC TC-3′ that includes the stop codon TAG). These fragments were then cloned into GZ41, resulting in the vector GZ44 (MYB75-SRDX) and GZ45 (*MYB75*-overexpression), respectively. GZ44 and GZ45 were transformed into Col as described above.

### Statistical analysis

One way analysis of variance (ANOVA), *t*-test, and χ^2^-test were performed using the SigmaStat® 3.1 software (Systat Software, Inc., USA). For the multiple comparison procedure in one way ANOVA, the Fisher LSD method was used, according to the user's manual provided by the vendor.

## Supporting Information

Table S1 Primers used in the cloning of the *OSU1* mutant gene
(0.10 MB DOC)

Table S2 Key parameters used in various RT-PCR reactions
(0.03 MB DOC)

Figure S1 *osu1-1* and *osu1-2* show similar hypersensitivity to the N-suppressed root growth in the absence of Suc (0C). Primary root lengths were measured after 7 days of vertical growth on agar-solidified media. The average of 7–8 seedlings is shown, with the bar representing the SD. Statistical analysis by one way ANOVA; the asterisk (*) above the column indicates a significant difference (p<0.05) between *osu1-1* or *osu1-2* and wild-type (WT) under the same C/N condition.
(1.78 MB TIF)

Figure S2 *osu1-1* and *osu1-2* show very similar seed germination kinetic profiles as wild-type. Seeds were sown on four representative C/N conditions (0.5C/0.05N, 0.5C/4N, 15C/0.05N, and 15C/4N) and cold-treated for two days before transfer to an incubator with 16 hour light/8 hour dark at room temperature. Germination was scored every two hours, and no seeds germinated before the 22nd hour after incubation. The data shows the average and the SD bar of three replicates, each with about 40 seeds. WT, wild-type.
(2.24 MB TIF)

Figure S3 Gene tree analysis of the OSU1-related puative methyltransferase family. A gene tree for all of 29 members of OSU1-related putative methyltransferases (shown on the left) was constructed, using the IAMT1 (encoded by At5g55250) as an outgroup, to analyze the relationships between each member and group. The protein sequences were aligned using the ClustalW 1.8 multiple sequence alignment tool (http://searchlauncher.bcm.tmc.edu/multi-align/multi-align.html). The alignment result was then used to generate a tree file in the PHYLIP format, and a tree was generated using the rectangular cluster algorithm through the web tool of TreeTop-Phylogenetic Tree Prediction (http://www.genebee.msu.su/services/phtree_reduced.html), with bootstrap values provided. Shown on the right is the seven amino acid motif, WVMNVVP, for each of 29 members, with the arrow indicating the N560Y mutation site in the *osu1-2* allele.
(0.69 MB TIF)
