# Supplementary material for: The OSU1/QUA2/TSD2-Encoded Putative Methyltransferase Is a Critical Modulator of Carbon and Nitrogen Nutrient Balance Response in Arabidopsis
Source: PLoS One. 2008 Jan 2;3(1):e1387. doi: 10.1371/journal.pone.0001387 (PMC2148111; doi:10.1371/journal.pone.0001387)
Supplement: Table S1 — Primers used in the cloning of the OSU1 mutant gene (0.10 MB DOC) [file pone.0001387.s001.doc]

**Table S1.** **Primers used in the cloning of the *OSU1* mutant gene.**

| Mapping | BAC clones | Name of markers | Name of primers ^b^ | Primer sequences (5'→3') |
| --- | --- | --- | --- | --- |
|  |  | NGA111 | NGA111S | TGTTTTTTAGGACAAATGGCG |
|  |  |  | NGA111A | CTCCAGTTGGAAGCTAAAGGG |
|  | F28K19 | CER451736 (9) ^a^ | F28K19M1S | GTTCTCTCTCAGATTCATCTTC |
|  |  |  | F28K19M1A | CAGAGGAAGTAGAATATATTGGC |
|  | T11I11 | 470559 (4) ^a^ | T11I11M1S | GCGTTAAATGATGTACGATCAGGT |
|  |  |  | T11I11M1A | GTCTGCCGGAAAAACATACTATAG |
|  |  | 470560 (1) ^a^ | T11I11M2S | CCATTAGATTTAAGTCACATGTGG |
|  |  |  | T11I11M2A | AATCCGCCGATGTGTCGGCCAGAG |
|  | F3F9 | 470251 (2) ^a^ | F3F9M5S | GCACTCAATCATATTGTTAAAACG |
|  |  |  | F3F9M5A | ATCCGAAAGTCAAGAGCCTCTTCG |
|  |  | 470249 (2) ^a^ | F3F9M4S | CCTCCTCATCCAAAACTATACCAA |
|  |  |  | F3F9M4A | CCTTGTGCTGGATAAGGTATGCAC |
|  |  | 470243 (6) ^a^ | F3F9M2S | TCCATTATGGTTTCTGGTCAACTG |
|  |  |  | F3F9M2A | TCTAGTCGTTACGTCACACCACTC |
|  | T30F21 | CER460363 (7) ^a^ | T30F21M1S | ACGGGTCATAATAGGTTAACATGA |
|  |  |  | T30F21M1A | CTAGATCCAACGGTTGAGATAGAG |
|  | YUP8H12R | CER451530 (14) ^a^ | YUP8H12RM2S | TGAATCATACTAATACAGAAGGAA |
|  |  |  | YUP8H12RM2A | ATTACGAGTTGTCTAATTTCTCCG |
|  | F23A5 | CER464729 | F23A5M1S | GTTTACGATCATCGTCTAGATAG |
|  |  |  | F23A5M1A | AGGAACTTGGAGCCAAACGCC |
| Sequencing | Gene ID | Positions on Chromosome 1 | Name of primers | Primer sequences (5'→3') |
|  | At1g78170 | 29419356 | GZP35 | CTATCTGCTCATTTCCTCACACG |
|  |  | 29419972 | GZP36 | GCATGATCGTTCATAGAAGCAGAG |
|  | At1g78180 | 29421946 | GZP37 | CGACTCTATCTCCTCCAAATGCTC |
|  | At1g78190 | 29423199 | GZP38 | ACCTGCTATAGCACCGTACATGAG |
|  |  | 29422876 | GZP39 | GGTTGTTCTCCCTCTACAAGGGTC |
|  |  | 29423984 | GZP40 | TCGCAAGTCCATTAGGGTACATCG |
|  | At1g78200 | 29425252 | GZP41 | GCGGAATTGAACCCAACTTAGGGT |
|  |  | 29426330 | GZP42 | CTTCGAAATACCGTCACTAGCCAG |
|  |  | 29426680 | GZP43 | ATAGTCCTATTCAAACTCATGTTC |
|  | At1g78210 | 29429294 | GZP44 | ATCCCCCAACCGATACCAGAGAAC |
|  |  | 29428068 | GZP45 | GGCTTTGATCAGTTCTCTCTTCTC |
|  |  | 29427621 | GZP46 | CTGAGCAACGAAGTGGTAATGTG |
|  | At1g78220 | 29432226 | GZP47 | GAGATCTGCTTCGACACATAATTG |
|  |  | 29431231 | GZP48 | CAGCATCAGAACCAAACTCTGCCA |
|  |  | 29431540 | GZP49 | GGAAGACTCGAAAGGAAACGACC |
|  |  | 29430441 | GZP50 | GAACCAAAGCACAAAGCCGAGAG |
|  | At1g78230 | 29434942 | GZP51 | CTCCGTGTATTGGACGCGGATAC |
|  |  | 29436053 | GZP52 | CCAGAGGGAAGTATCTGCATCGAC |
|  |  | 29435806 | GZP53 | ACAATGACGAGGATGAAGGAGGTC |
|  |  | 29436958 | GZP54 | TGCTGATCTTGTTCCCAGCTAGG |
|  |  | 29436701 | GZP55 | CCTAAGGGCCTCCACGCGTTGAAC |
|  |  | 29437580 | GZP56 | CATGTTTCCGAGAACCGGATCAAC |
|  | At1g78240 | 29440811 | GZP57 | GCGTGATCTCGAGTTTCAGAGATC |
|  |  | 29439673 | GZP58 | CAATAGATGTGCCCCAAAGCTACC |
|  |  | 29439927 | GZP59 | GATATCTTTCAGGTCAGCATCTCC |
|  |  | 29438785 | GZP60 | CTCAGCATCCTCTCCAAGCACCTC |
|  |  | 29439078 | GZP61 | ACCTGGAGTGGGCCCTTCTGTGTG |
|  |  | 29437943 | GZP62 | TCTTCCTCCTTTCTTCCCCGTATC |
|  | At1g78250 | 29448662 | GZP63 | CTTCGGACCTGGTGGGCTTCTTAG |
|  |  | 29449094 | GZP64 | GAGAATTCCAACTGGTCTTAATCG |

Notes: *^a^* The numbers after the marker names indicate the number of recombinants (see Figure 6A). The *osu1* mutation is mapped to a region between the markers, 470560 on the BAC clone T11I11 and 470251 on the BAC clone F3F9. *^b^* In the mapping category, primer name ending with "S" denotes "sense", while "A" denotes "antisense". In the sequencing category, the first one is the sense primer and the second one is the antisense primer.
