# Supplementary material for: The OSU1/QUA2/TSD2-Encoded Putative Methyltransferase Is a Critical Modulator of Carbon and Nitrogen Nutrient Balance Response in Arabidopsis
Source: PLoS One. 2008 Jan 2;3(1):e1387. doi: 10.1371/journal.pone.0001387 (PMC2148111; doi:10.1371/journal.pone.0001387)
Supplement: Table S2 — Key parameters used in various RT-PCR reactions (0.03 MB DOC) [file pone.0001387.s002.doc]

**Table S2.** **Key** p**arameters used in various RT-PCR reactions**

| PCR types | Genes | Primers | Annealing Temperature (°C) | Number of cycles |
| --- | --- | --- | --- | --- |
| Regular PCR | *MYB75* | GZP7 and GZP8 | 65 | 39 |
|  | *OSU1* | ZZP87 and ZZP88 | 54 | 35 or 39 |
| Real-time PCR | For *MYB75*, *MYB90*, *ASN1* and *OSU1* | See “Materials and  Methods” in the text | 60 | 45 |
