# Supplementary figures and images for: The OSU1/QUA2/TSD2-Encoded Putative Methyltransferase Is a Critical Modulator of Carbon and Nitrogen Nutrient Balance Response in Arabidopsis
Source: PLoS One. 2008 Jan 2;3(1):e1387. doi: 10.1371/journal.pone.0001387 (PMC2148111; doi:10.1371/journal.pone.0001387)

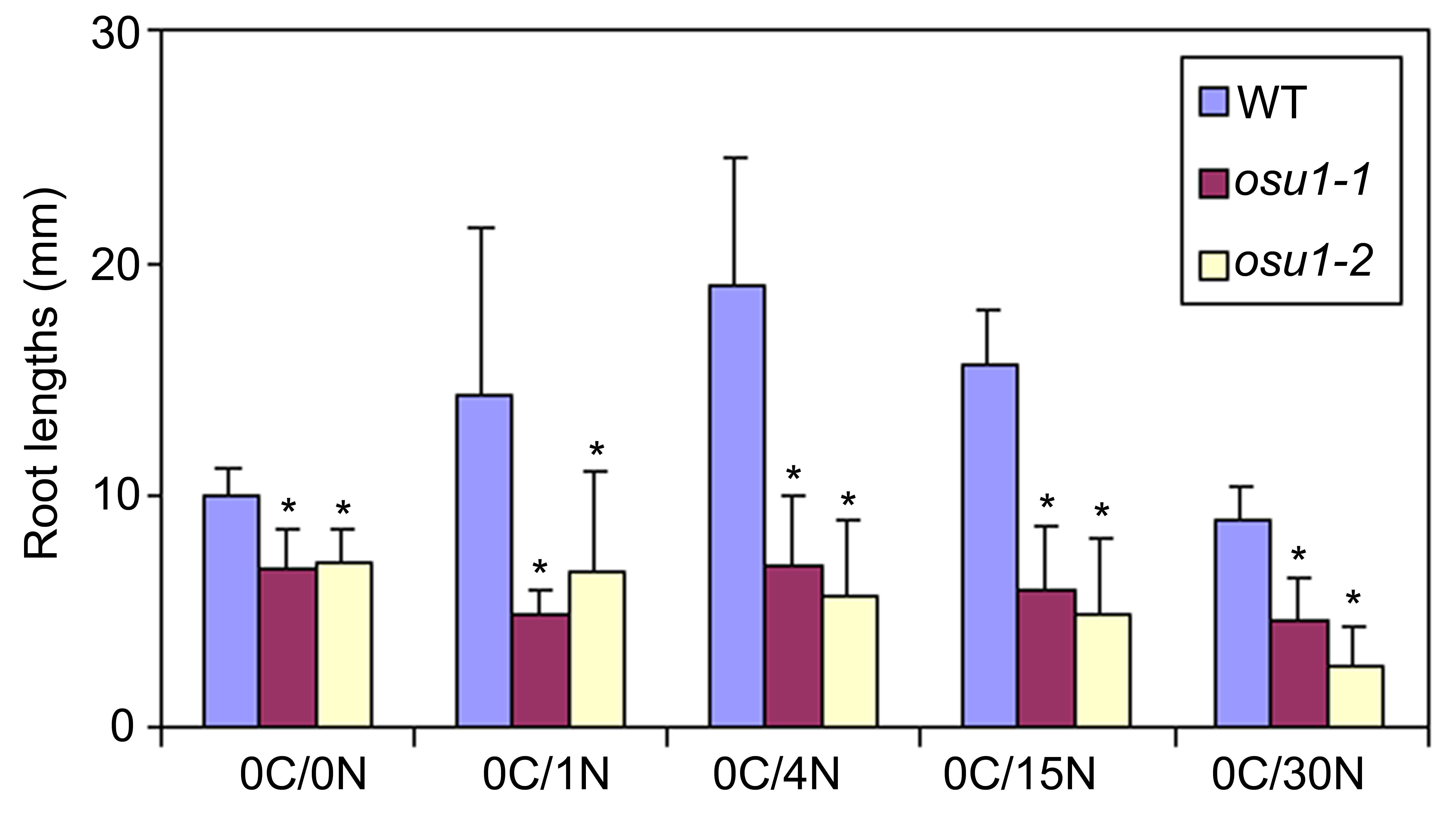

Supplement: Figure S1 — osu1-1 and osu1-2 show similar hypersensitivity to the N-suppressed root growth in the absence of Suc (0C). Primary root lengths were measured after 7 days of vertical growth on agar-solidified media. The average of 7–8 seedlings is shown, with the bar representing the SD. Statistical analysis by one way ANOVA; the asterisk (*) above the column indicates a significant difference (p<0.05) between osu1-1 or osu1-2 and wild-type (WT) under the same C/N condition. (1.78 MB TIF) [file pone.0001387.s003.tif]

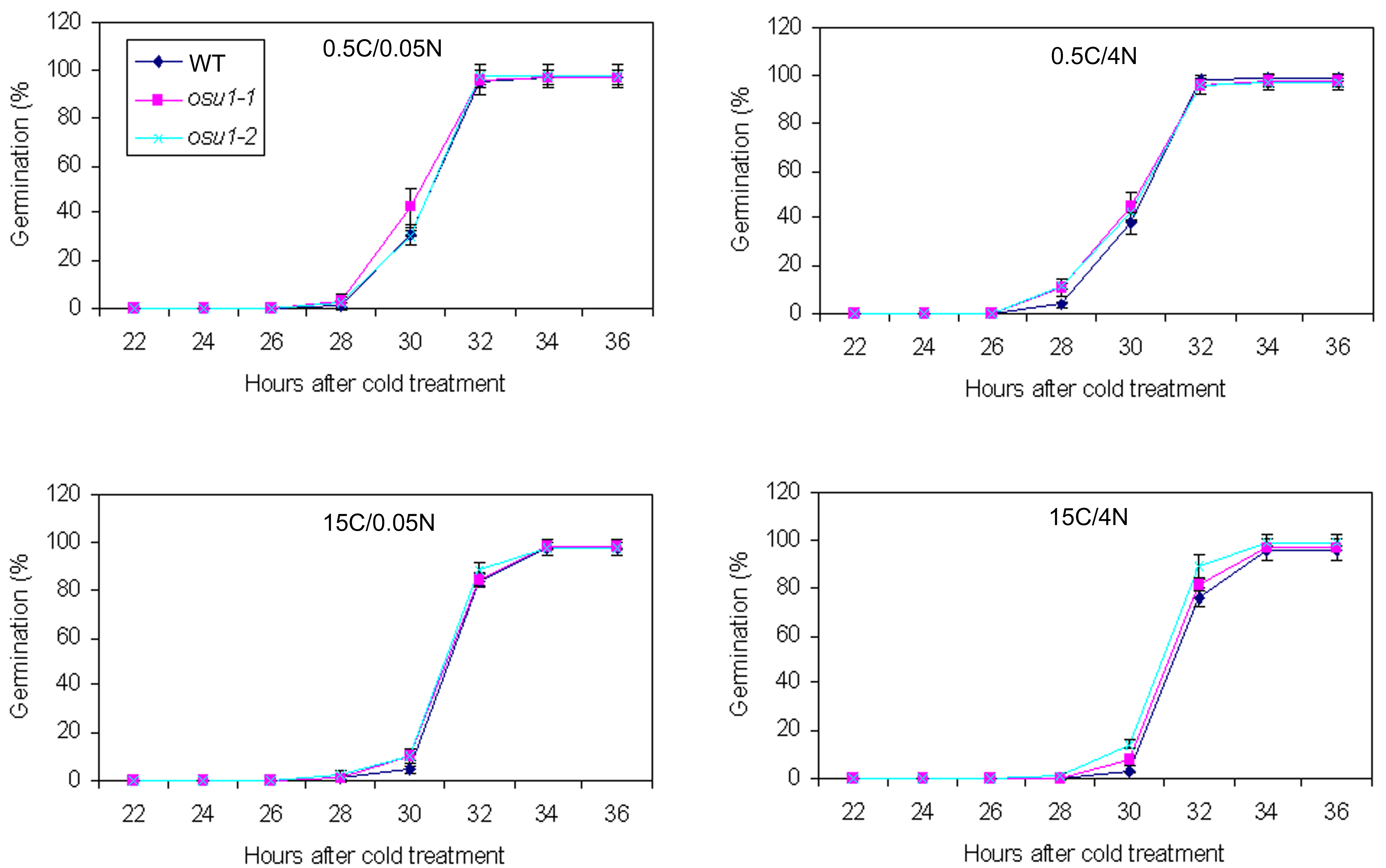

Supplement: Figure S2 — osu1-1 and osu1-2 show very similar seed germination kinetic profiles as wild-type. Seeds were sown on four representative C/N conditions (0.5C/0.05N, 0.5C/4N, 15C/0.05N, and 15C/4N) and cold-treated for two days before transfer to an incubator with 16 hour light/8 hour dark at room temperature. Germination was scored every two hours, and no seeds germinated before the 22nd hour after incubation. The data shows the average and the SD bar of three replicates, each with about 40 seeds. WT, wild-type. (2.24 MB TIF) [file pone.0001387.s004.tif]

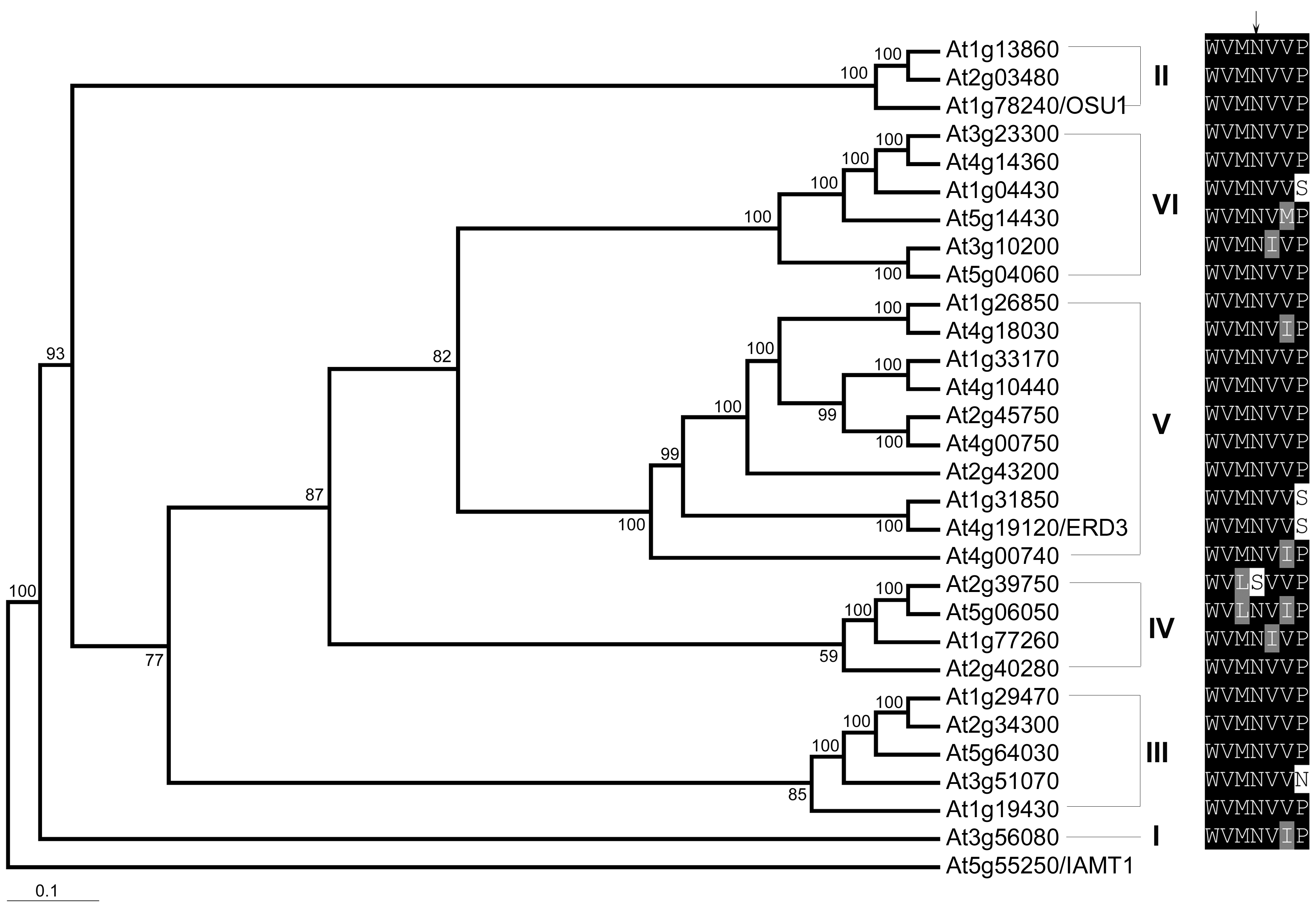

Supplement: Figure S3 — Gene tree analysis of the OSU1-related puative methyltransferase family. A gene tree for all of 29 members of OSU1-related putative methyltransferases (shown on the left) was constructed, using the IAMT1 (encoded by At5g55250) as an outgroup, to analyze the relationships between each member and group. The protein sequences were aligned using the ClustalW 1.8 multiple sequence alignment tool (http://searchlauncher.bcm.tmc.edu/multi-align/multi-align.html). The alignment result was then used to generate a tree file in the PHYLIP format, and a tree was generated using the rectangular cluster algorithm through the web tool of TreeTop-Phylogenetic Tree Prediction (http://www.genebee.msu.su/services/phtree_reduced.html), with bootstrap values provided. Shown on the right is the seven amino acid motif, WVMNVVP, for each of 29 members, with the arrow indicating the N560Y mutation site in the osu1-2 allele. (0.69 MB TIF) [file pone.0001387.s005.tif]
